# Prospective Associations of Dietary and Nutrient Patterns with Fracture Risk: A 20-Year Follow-Up Study

**DOI:** 10.3390/nu9111198

**Published:** 2017-10-31

**Authors:** Yohannes Adama Melaku, Tiffany K. Gill, Sarah L. Appleton, Anne W. Taylor, Robert Adams, Zumin Shi

**Affiliations:** 1 Adelaide Medical School, The University of Adelaide, Adelaide, SA 5005, Australia; tiffany.gill@adelaide.edu.au (T.K.G.); anne.taylor@adelaide.edu.au (A.W.T.); zumin.shi@adelaide.edu.au (Z.S.); 2 Department of Human Nutrition, Institute of Public Health, University of Gondar, Gondar 196, Ethiopia; 3 The Health Observatory, Discipline of Medicine, The Queen Elizabeth Hospital Campus, University of Adelaide, Woodville, SA 5011, Australia; sarah.appleton@adelaide.edu.au (S.L.A.); robert.adams@adelaide.edu.au (R.A.); 4 Freemason’s Centre for Men’s Health, Discipline of Medicine, The University of Adelaide, Adelaide, SA 5005, Australia

**Keywords:** dietary pattern, nutrient pattern, fracture, China Health and Nutrition Survey

## Abstract

Studies on long-term exposure to foods/nutrients and its associations with fracture risk are scarce. Using data from the China Health and Nutrition Survey (CHNS), we determined the prospective association of dietary and nutrient patterns with fractures. Data from 15,572 adults aged ≥18 years were analyzed. Fracture occurrence was self-reported and dietary intake data were collected using a 24-h recall method for three consecutive days, for each individual across nine waves (1989–2011). We used cumulative and overall mean, recent and baseline dietary and nutrient exposures. Hazard ratios (HR) were used to determine the associations. Two dietary (traditional and modern) and two nutrient (plant- and animal-sourced) patterns were identified. After adjusting for potential confounders, study participants in the third tertiles (highest intake) of the modern dietary and animal-sourced nutrient patterns’ cumulative scores had a 34% (HR = 1.34; 95% CI: 1.06–1.71) and 37% (HR = 1.37; 95% CI: 1.08–1.72) increase in fracture risks compared to those in the first tertiles, respectively. While the overall mean factor scores of dietary and nutrient patterns had a similar (or stronger) pattern of association as the cumulative scores, no association between recent and baseline scores and fracture was found. Greater adherence to a modern dietary and/or an animal-sourced nutrient pattern is associated with a higher risk of total fractures. This suggests that a modern animal based diet is related to bone fragility. A repeated three-day 24-h recall dietary assessment provides a stronger association with fracture compared to a recent or baseline exposure.

## 1. Introduction

Lifestyle and behavioral factors are associated with fracture risk [1,2]. Of the lifestyle and behavioral factors, diet is of a particular significance [3,4,5]. Previous studies have generally focused on the associations between individual diets or nutrients with fractures [6,7,8,9,10]. This approach does not consider other food items or nutrients that could have a potential influence on fracture risk; and the interactions of food items or nutrients are ignored resulting in a biased (confounded) association with fracture risk. Realistically, people do not consume individual foods or nutrients but rather a mixture of foods with multiple nutrients. Furthermore, bone physiology is not dependent on individual nutrients, thus these combinations provide a further challenge for clinical and public health recommendations to improve bone strength.

Studies have shown inconsistent findings on the association between dietary patterns and fracture risks [4,11,12,13]. In terms of nutrient patterns, to the best of our knowledge, with the exception of one study [14], no other studies have investigated the association with fracture risks. A thorough investigation of an association between patterns of nutrient and food intakes over the long term, and fractures, is essential as bone is a complex structure composed of multiple nutrients. In addition, diet and/or nutrients that are associated with muscle mass or strength could also determine fracture risks [15]. Focusing on the overall dietary and nutrient patterns assists dietary counseling and recommendations for individuals and population groups and this approach can also detect a potential positive impact of minimal changes across foods or nutrients, rather than a major change in a few food or nutrient groups on health outcomes, which might result in a better compliance of dietary recommendations [16]. In this study, we aimed to assess prospective associations between long term dietary and nutrient patterns and fracture risk among adults (18 years and above) using the Chinese Health and Nutrition Survey (CHNS).

## 2. Materials and Methods

### 2.1. Study Design and Population

**Figure 1:**
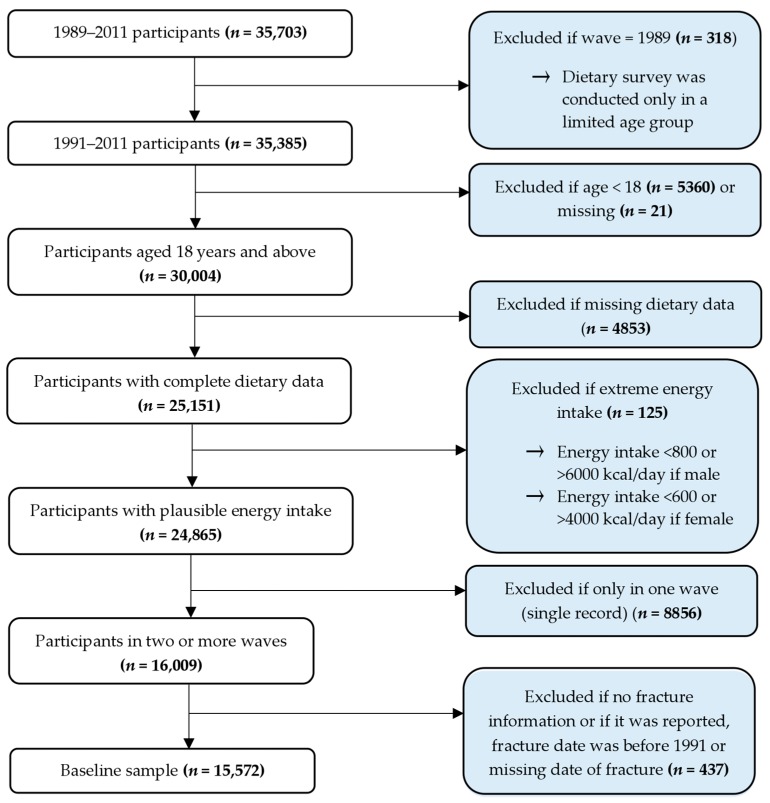
Sampling description.

We used longitudinal data from the CHNS, which is an open prospective cohort study and represents nine provinces of China [17]. There were nine waves (two to three years apart) of data collection between 1989 and 2011. A multistage random-cluster sampling technique was used to select households in the study. All members of the selected households were eligible to be included in the study. Between 1989 and 2011, 35,703 study participants were involved in at least one study wave. After excluding those who were not eligible, the baseline sample was 15,572 (Figure 1). The response rates based on those who participated in previous waves staying in the subsequent survey were around 88%. However, the response rate out of the participants included at baseline (1989) and remained in 2006 was more than 60% [18]. The CHNS was approved by the institutional review committees of the University of North Carolina (Chapel Hill, NC, USA) and the National Institute of Nutrition and Food Safety (Beijing, China). Prior to the survey, informed consent was obtained from all participants.

### 2.2. Outcome Variable

Fracture was self-reported in each wave by the study participants for a question “Have you ever had fracture?” along with age when the first fracture occurred. To determine the calendar year of fracture, first we calculated the difference between the current age (at the interview) and age at first fracture. Then, we subtracted the age differences (in years) from the respective calendar years or waves (when the interview was conducted). This provided us the calendar year of the first facture. We assumed that the date of fracture was on 1 July of each year. In a previous large cohort study, a self-reported assessment of lifetime fractures, along with age at fractures, was found to be a feasible method to establish incident cases [19]. We excluded those participants who had the first fractures before the first interview date for each wave (when dietary data were collected) and those with less than 0.5 years of follow-up after the interviews.

### 2.3. Assessment of Dietary and Nutrient Intakes

Detailed descriptions of dietary measurements are provided elsewhere [20]. In short, dietary intake data were collected using a 24-h dietary recall method for three consecutive days at each wave for each individual. At the beginning and end of the three days, interviewers weighed/recorded all available and wasted foods at home. These data were linked and harmonized with the dietary recall data to determine individuals’ dietary intake levels. The Chinese Food Composition Table was used to analyze the food consumption data (g/day) and to determine the intake levels of nutrients. Foods and nutrients were categorized into 34 and 21 groups for further analysis, respectively.

### 2.4. Covariates

At each wave, data on socio-demographic, lifestyle, physical measurements and chronic conditions were collected. Individual level income was classified into tertiles (low, middle and high) at each wave. The highest level of education achieved was categorized into low (illiterate or primary school), medium (junior middle school) and high (high middle school or higher). Residency was classified into two categories (urban and rural) based on an urbanization index which is a composite of 12 components that included population and other socioeconomic characteristics [20]. Lifestyle factors included smoking, alcohol consumption and physical activity levels (PAL). We categorized smoking status as non-smokers and current smokers/ex-smokers. Frequency of alcohol consumption was categorized as “none”, “<1/week”, “1–2/week”, “3–4/week” and “daily”. PAL, in terms of metabolic equivalent of tasks (MET-hours per week), was determined based on self-reported job and leisure time activities, intensity and duration of the activities.

Height and weight were measured based on a protocol recommended by World Health Organization (WHO). Body mass index (BMI) was calculated as weight (kg) divided by the square of height (m). Hypertension was determined based on systolic (above 140 mmHg) and/or diastolic (above 90 mmHg) blood pressure measures, or having doctor diagnosed hypertension.

### 2.5. Statistical Analysis

Dietary and nutrient patterns were identified across the seven waves (1991–2009) by factor analysis using the principal component method. Eigenvalue (>1.5), scree plot, and interpretability of the factors were used to determine the number of dietary and nutrient patterns. Factor loadings (the correlation between each pattern and the food and nutrient groups) were calculated. Percentages of variances (the variations that were explained by the identified dietary and nutrient patterns) were also computed. For each dietary and nutrient pattern, factor scores were assigned across all study participants. Factor scores show the relative position of the study participants in each of the identified patterns reflecting adherence to the patterns. Pattern-specific factor scores are calculated as the sum of the products of the factor loading coefficients and standardized daily consumption of food and nutrient groups related with the pattern. The factor scores were orthogonally (varimax) rotated to create less correlation among the patterns and to facilitate their interpretability.

Based on the factor scores for the dietary and nutrient patterns, four approaches were used to determine the exposure levels (measured in scores) of dietary and nutrient patterns and assess the association between the patterns and fracture risk. The first approach was to calculate the cumulative exposure level. To represent the usual relative position (factor scores or adherence to the patterns) of the study participants in the factors [21], we calculated cumulative mean factor scores. The cumulative scores were calculated by summing factor scores and dividing by the number of waves contributing to the scores for each study participant. For example, for the second wave (1993), factor scores of the first wave (1991) were used; for the third wave (1997), an average of scores of waves one (1991) and two (1993) was used; and, for the fourth wave (2000), an average of factors of waves one, two and three (1997) was used. Correlations between cumulative scores of dietary and nutrient patterns were investigated with Spearman rank correlations.

The second approach was using the overall mean of the dietary and nutrient pattern scores. The overall mean was calculated by summing factor scores until the wave just prior to the fracture or censoring occurred and dividing by the number of waves contributing to the scores for each study participant. The third and fourth approaches used the recent and baseline factor scores. The participants were then allocated into tertiles (first (lowest intake); second; and third (highest intake) tertiles) based on the factor scores.

Chi-square (categorical variables), analysis of variance (ANOVA) (normally distributed continuous variables) and Kruskal–Wallis (continuous but not normally distributed) tests were used where appropriate to compare the differences in proportions, means and medians of the groups at baseline. Time to the incident event was determined as the time from enrolment to the first occurrence of incident fracture. Follow-up was censored at the date of the outcome event, end of follow-up, date of outmigration, or date of death whichever came first.

We calculated the incidence rate (per 1000 person-years) of fractures by tertiles of dietary and nutrient patterns and the log-rank test was used to investigate the differences. Nelson–Aalen cumulative hazard estimates were calculated by tertiles of the patterns across the follow-up time. To assess the associations of dietary and nutrient patterns with incident fractures, hazard ratios (HRs) for fractures and tertiles of the cumulative and overall mean, recent, and baseline factor scores were determined using Cox proportional hazard regression models. The first tertile was used as reference category. Three models were used to determine HRs: Model 1 adjusted for age, sex and daily energy intake; Model 2 additionally adjusted for education status, income, alcohol intake, residency and PAL; and Model 3 was further adjusted for BMI and hypertension. Using Model 3, we also conducted stratified analyses using age group (age < 50 and ≥ 50) and sex to explore and compare the associations in the respective groups. We tested interactions between dietary and nutrient patterns, other covariates and fracture risks using multiplicative terms in the last model (Model 3). The assumption of proportionality was tested by including time-dependant covariates in the final models and was valid for all analyses. To assess the quality of models (Model 3), we determined Akaike’s information criterion (AIC). We estimated the absolute risk differences for fractures between the third and first tertiles and the number of individuals needed to get one fracture case as a consequence of being in the third tertiles of dietary and nutrient patterns. Participants were also jointly classified across tertiles of dietary and nutrient patterns and used in the Cox regression (Model 3). Statistical analyses were performed using Stata version 14 (Stata Corporation, College Station, TX, USA). All *p* values are two-sided.

## 3. Results

### 3.1. Baseline Characteristics

Baseline characteristics of the study participants are shown in Table 1. The study participants were followed for 20.2 years (median follow-up time = 8.9 years), which equates to a total of 162,416.3 person-years.

### 3.2. Dietary and Nutrient Patterns

Figure 2 depicts the identified dietary and nutrient patterns and factor loadings of food groups. Two dietary patterns were identified. Whereas the first pattern (traditional) was characterized by high intake of rice, pork, fish, poultry, dry tofu, beef, fresh vegetables and offal, the second pattern (modern) was characterized by high intake of fruits, milk, cake, fast foods, eggs, soy milk and deep fried products. The two patterns explained 11.9% of variance. Two nutrient patterns (plant- and animal-sourced) were determined. The two nutrient patterns explained 59.1% of nutrient intake variance. The correlations between the traditional dietary pattern and the plant- and animal-sourced nutrient pattern cumulative scores were −0.051 and 0.127, respectively; and between the modern dietary pattern and plant- and animal-sourced nutrient patterns were −0.306 and 0.462, respectively (*p* ≤ 0.0001) (Table S1).

Consumption patterns of selected food and nutrient groups across the tertiles of dietary and nutrient patterns are also shown in Table 2. Overall, the consumption of milk was very low (5.8 mL/day). There was a significant reduction of calcium, fiber and vitamin C intake across the tertiles of animal-sourced nutrient patterns (*p* < 0.001).

### 3.3. Dietary and Nutrient Patterns and Fracture Rate

During the follow-up, there were 649 incident cases of fractures (males = 311 and females = 338). The rate of fracture was 4.0 (95% CI: 3.7–4.3) per 1000 person-years (Table 3). While males (3.8 per 1000 person-years) below 50 years of age had a higher fracture rate compared to their female (2.9 per 1000 person-years) counterparts, the reverse (2.8 (males) vs. 6.4 (females) per 1000 person-years) was found for those 50 years and over (Table S2). Nelson–Aalen cumulative hazard estimates by tertiles of dietary and nutrient patterns are depicted in Figures S1 and S2.

After adjusting for potential confounders (socio-demographic, lifestyle and chronic conditions), participants in the third tertile of modern dietary pattern scores (cumulative mean) had a 34% increased fracture risk (HR = 1.34; 95% CI: 1.06–1.71) compared to those in the first tertile (Table 4). The absolute risk increase was 0.30% (95% CI: 0.06–0.54) and a number needed to have one fracture case was 339 (95% CI: 188–1785). Participants in the second (HR = 1.29; 95% CI: 1.04–1.60) and third tertiles (HR = 1.37; 95% CI: 1.08–1.72) of animal-sourced nutrient pattern cumulative scores had a higher risk of fracture compared to those in the first tertile with an absolute risk increase of 0.31% (95% CI: 0.08–0.55) and a number needed to have one case of fracture of 321 (95% CI: 184–1285).

In joint classification of study participants according to adherence to different dietary and nutrient patterns, the risk of fracture was higher with higher adherence to the modern pattern in each stratum of traditional dietary and animal-sourced nutrient patterns. We found a 32% (95% CI: 52–1%) reduction of fracture rate for those who had simultaneous category of lowest adherence to plant- and animal-sourced nutrient patterns (Figures S3–S5).

The estimates of association between tertiles of overall mean factor scores and fracture provided a similar pattern to the cumulative factor scores of dietary and nutrient patterns. However, there was no association between the recent and baseline factor scores of dietary and nutrient patterns and fracture (Table 4). There were no interactions between the dietary/nutrient patterns, other covariates and fracture risk (data not shown). Stratified analyses by age and sex are provided in Table S3.

## 4. Discussion

Two dietary (traditional and modern) and two nutrient (plant- and animal-sourced) patterns were identified using the CHNS data. In this analysis, with up to 20 years of follow-up, we found that a greater adherence to a modern dietary (characterized by high intake of fruits, milk, cake, fast foods, eggs, soy milk and deep fried products) and/or animal-sourced nutrient patterns (high intake of protein, fat, vitamins A, B2 and E, and low intake of potassium, calcium, magnesium and vitamin C) was prospectively associated with increased fracture risks among adults. In this study, we demonstrated that, compared to a single three-day 24-h dietary assessment method (at baseline or recent), a repeated three-day 24-h dietary assessment provided a stronger estimate of the association with fracture risk as it reflected usual food intake more closely. This highlights the problem of using a baseline or a recent dietary exposure to estimate the association between diet and fracture in cohort studies which could provide a biased estimate leading to a wrong conclusion.

### 4.1. Comparison with Other Studies

Studies among men and women in the United States of America (USA) and Sweden found a lower risk of hip fractures among those who had higher adherence to a Mediterranean diet [4,22]. Studies have also shown the benefit of vegetables, legumes and whole grains as part of a healthy dietary pattern in maintaining bone mass and preventing osteoporotic fractures [4,11,13]. Thus, a low intake of vegetables, legumes and whole grains could explain the positive association between the modern pattern and fracture in our study. In studies among Chinese populations, it has also been found that favorable dietary patterns (high intake of fruit, vegetable, nuts, soy and seafood) were inversely associated with hip fractures [3,5]. It is of note however that the intake of milk in our study was highly correlated with the modern dietary pattern, although milk is largely considered to be an essential part of a favorable dietary pattern for bone health in many studies [23,24]. However, the overall milk consumption among the study participants in the current study was very low (5.8 mL/day) which may contribute to the findings.

### 4.2. Potential Mechanisms

The increased risk of fracture associated with higher adherence to modern dietary pattern could be explained by the direct effect of food groups on bone mass and/or indirect influence on skeletal muscle. Previous studies have shown inverse association between modern and processed dietary patterns with bone mass [25,26], which consequently lead to a higher risk of osteoporotic fractures. These dietary patterns are mainly loaded with a high intake of suboptimal diets, such as energy-dense or nutrient-poor foods [27,28], which have been associated with reduced bone mass. On the other hand, risk factors for fractures are multifaceted and might not necessarily be associated with low bone mass [29].

Because of the fact that fractures could be related with falls as a result of a lower muscle mass/strength [15], diets could have also indirect effect on fracture risk through their impact on muscle. For instance, a “western” type of dietary pattern (characterized by high intake of red meats, potato, gravy and butter) was negatively associated with muscle strength [30]. Similarly, a higher risk of fall-related fractures was reported among elderly Japanese who had a higher adherence to a “meat” based dietary pattern [31]. On the other hand, a higher adherence to Mediterranean diet was associated with a lower risk of frailty [32].

The effect of dietary patterns on body acid-base balance [33] and inflammation [34] could be another possible indirect pathway through which dietary patterns affect bone mass, and eventually fracture risk. In people with a low intake of calcium, a higher dietary acid load was associated with lower bone mineral density [35]. A higher net endogenous acid production would result in decreased extracellular pH, creating an acidic environment. This phenomenon could facilitate the release of calcium from bone matrix in order to buffer the higher acid levels. In addition, it might also increase osteoclast and decrease osteoblast activities (i.e., facilitated bone resorption), eventually resulting in increased calcium excretion [36] and reduced bone mass [37]. However, the epidemiological evidence remains inconclusive, pending further investigation [38]. In recent studies, pro-inflammatory diets were associated with a lower bone mineral density [39] and an increased risk of hip fractures in women [34]. In addition to non-nutritive substances in dietary patterns, the combination of nutrients may take a major role on fracture risks directly through affecting bone mass or indirectly through increasing body acid and/or inflammation.

In the current study, we found that a higher adherence to an animal-sourced nutrient pattern was associated with a higher risk of fractures. The factor scores of this nutrient pattern were also positively and moderately correlated with the scores of the modern dietary pattern. A nutrient pattern characterized by a high intake of calcium, phosphorous, vitamin B12, proteins and saturated fats was related with a lower risk of wrist and hip fracture among French older people (aged 65 and over) [14]. The difference in calcium, phosphorous, protein and fat content of nutrient patterns associated with the fracture risk in this and our current studies could be explained by the general difference in population groups, such as, age, eating habit and race. Protein (93.5 g/day) and fat (51.9 g/day) intake was found to be higher among study participants in the third tertile of the animal-sourced nutrient pattern compared to those in the first tertile in the current study. Although the evidence on the effect of high protein intake on bone mass is inconsistent [40,41,42], it is believed that higher protein intake can lead to calciuria [43], causing bone resorption. In addition, a low-fat diet was associated with reduced risk of multiple falls among postmenopausal women [13].

Inflammation of the body can also increase bone resorption and decrease bone formation through various pathways [44] making the bone more susceptible to low-trauma fracture. A study among Australian men found that animal-sourced nutrient pattern was associated with enhanced inflammatory markers [45]. In another prospective cohort study in the USA, increased inflammatory markers were positively associated with incident fracture risks among older men and women [46], further supporting the fact that the pro-inflammatory effect of the animal-sourced nutrient pattern in the current study may explain the positive association with fracture risks. In line with our study, a higher adherence to an inflammatory diet (mainly containing high animal-sourced nutrients) was associated with an increased risk of hip fracture in younger women (less than 63 years) [34]. This suggests that clinical and public health interventions and strategies should consider dietary approaches in prevention of fractures among high risk adults.

Interactions between dietary and nutrient patterns and other covariates in predicting fracture risk were not found. Stratified analyses by sex and age, however, gave a slightly different result. Modern dietary and animal-sourced patterns were significantly associated with fracture risks in males, but not in females (although the association remained in the same direction). In females, a higher adherence to a plant-sourced nutrient pattern was significantly associated with an increased risk of fractures. Although the direction of association remained the same in those aged less than 50 years, the association between the modern dietary pattern and fracture risk was significant only in those aged 50 years or over. The difference in the associations may be attributable to differences in body physiology (including bone physiology), hormonal changes, change in dietary habit and/or a low number of fracture cases in the stratified analyses. In addition, causes (low-energy vs. high-energy injury) of fractures might be different in different age categories and sexes. In this regard, our study showed that the risk of fracture was higher in males than females at a younger age while the vice versa was found for the older age bracket. This may indicate that most of the fractures in young males could be due to high-energy traumas. Further research is warranted in this regard. An animal-sourced nutrient pattern remained significantly associated with fracture risks in both age brackets (<50 and ≥50 years and over).

### 4.3. Dietary Exposure Measurement

Our study also demonstrates that the identification of dietary risks of a disease outcome (in this case fracture) using a repeated 24-h dietary recall method is likely to provide a stronger estimate of an association compared to a baseline or recent dietary exposure. Dietary data collected using a repeated three-day 24-h recall method give a better picture of the usual food intake compared to a baseline or a recent dietary exposure using a single three-day 24-dietary assessment method, which eventually provides a stronger association estimate for a disease outcome [47]. In our study, use of recent or baseline dietary that rely on static eating behaviors exposure underestimated the associations. This is supported by a previous study which used multiple dietary measurements during a follow-up period to assess the effect of dietary fat on coronary heart disease. It was found that this approach provided a better estimate compared to baseline or recent dietary exposures [21].

It is important to note the following limitations of this study when interpreting the findings. First, data on fractures were self-reported. The dates of the fractures were determined based on participants’ recall of ages at which the fractures occurred, which may be impacted by a recall bias. In addition, the dates of fractures might not be accurate. However, this approach has previously been found to be a feasible alternative to hospital and X-ray records in determining relative fracture incidence across population subgroups, particularly for recent fractures, in a large cohort study [19]. Secondly, since fractures were not segregated into low- and high-energy injuries, it was not possible to determine the specific low-energy trauma fracture cases potentially due to a reduced bone mass. However, a study reported that most fracture cases (58%) in China (2014) were caused by low-energy injuries (Slip, trip, or fall) [48]. Body sites of fractures were also not reported in the survey—fractures of toe, finger, sternum, and clavicle are less likely to be linked with osteoporosis [49,50]. However, this method of fracture reporting has been used in a previous study [34] and in China the highest incidence rates of fracture occurred on tibia and fibula (0.76 fractures per 1000 people) and radius and ulna (0.63 per 1000 people) [48]. Thirdly, although we adjusted for potential confounders, residual confounding from unmeasured lifestyle variables (such as from duration of sleep [48,51]) is still possible. In addition, not being able to adjust for medication (such as psychoactive medications) and supplement (hormonal and dietary) use could potentially overestimate the associations. However, in China, the proportion of women using, for example, hormonal replacement therapy has previously been reported as being 2.1% [52]. Thus, the effect of this confounder may be small.

## 5. Conclusions

In summary, modern dietary and animal-sourced nutrient patterns are prospectively associated with fracture risk. This study highlights the important role of diet and nutrients in fracture risk among adults. Clinical and public health interventions that target increasing or maintaining bone mass and lowering fracture risks should take into account dietary approaches as important strategies at individual and population levels. Repeated measures of dietary exposure provide a stronger estimate in determining an association with a disease outcome. On the contrary, using a baseline or a recent exposure of dietary score to estimate the association between diet and a disease outcome in prospective studies could provide a biased estimate.

## Figures and Tables

**Figure 2:** Factor loadings of food groups and nutrients to dietary and nutrient patterns (the color gradation reflects how big and in which direction was the correlation between the food groups and nutrients, and the patterns. Deep green color refers to a relatively higher correlation (higher intake) of the food groups and nutrients with the dietary and nutrient patterns, respectively. Deep red color refers to a relatively lower correlation (a lower intake) of the food groups and nutrients with the patterns).

**Table:** 

Food Groups	Dietary Patterns and Factor Loadings		Nutrients	Nutrient Patterns and Factor Loadings	
	Traditional	Modern		Plant-Sourced	Animal-Sourced
Rice	0.71	−0.40	Potassium	0.93	−0.08
Pork	0.48	0.21	Phosphorus	0.93	0.26
Fish	0.41	0.19	Magnesium	0.93	0.08
Poultry	0.29	0.21	Zinc	0.87	0.32
Dry tofu	0.29	0.03	Calcium	0.87	−0.17
Beef	0.24	0.16	Iron	0.86	0.19
Fresh vegetable	0.24	−0.20	Copper	0.82	0.25
Offal	0.24	0.03	Fiber	0.79	0.08
Mushroom	0.16	0.29	Manganese	0.78	0.11
Spirit	0.12	0.10	Vitamin C	0.78	−0.26
Shrimp	0.11	0.22	Carbohydrate	0.73	0.17
Nuts	0.09	0.23	Niacin (vitamin B3)	0.72	0.39
Beer	0.09	0.23	Thiamine (vitamin B1)	0.70	0.37
Fruit	0.08	0.44	Sodium	0.25	0.11
Salted vegetable	0.07	−0.21	Riboflavin (vitamin B2)	0.22	0.79
Milk	0.05	0.40	Protein	0.61	0.70
Beverage	0.04	0.12	Fat	0.08	0.66
Yoghurt	0.03	0.16	Selenium	0.30	0.52
Sugar	0.03	0.11	Vitamin E	0.34	0.51
Wine	0.02	0.03	Vitamin A	−0.07	0.47
Milk powder	0.02	0.06	Folate	−0.04	0.11
Lamb	0.01	0.18			
Fresh bean	0.00	0.03			
Cake	−0.02	0.31			
Legume	−0.02	−0.11			
Fast food	−0.03	0.40			
Eggs	−0.03	0.44			
Tofu	−0.05	0.05			
Soy milk	−0.07	0.42			
Bean thread noodle	−0.09	0.07			
Tubers	−0.19	−0.13			
Deep fried products	−0.20	0.41			
Whole grain	−0.47	−0.04			
Wheat	−0.73	0.07			

**Table 1:** Baseline socio-demographic characteristics across tertiles of dietary and nutrient patterns among adults 18 years and above, the China Health and Nutrition Survey.

Characteristics	Overall		T1	T2	T3	*p* Value	T1	T2	T3	*p* Value
	Category	Value	Traditional Dietary Pattern				Modern Dietary Pattern			
*N*		15,572	5476	5164	4932		6019	4796	4757	
Sex ^#^	Male	7627 (49.0%)	2613 (47.7%)	2192 (42.4%)	2822 (57.2%)	<0.001	3000 (49.8%)	2213 (46.1%)	2414 (50.7%)	<0.001
Age in years, median (IQR) ^$^		37.6 (27.5, 51.1)	37.9 (27.5, 51.7)	38.6 (27.9, 53.3)	36.3 (26.7, 47.9)	<0.001	36.5 (26.8, 48.4)	37.7 (27.2, 51.5)	39.2 (28.6, 53.5)	<0.001
Income ^#^	Low	4537 (29.1%)	2010 (36.7%)	1335 (25.9%)	1192 (24.2%)	<0.001	2409 (40.0%)	1481 (30.9%)	647 (13.6%)	<0.001
	Medium	5083 (32.6%)	1739 (31.8%)	1728 (33.5%)	1616 (32.8%)		2066 (34.3%)	1651 (34.4%)	1366 (28.7%)	
	High	5842 (37.5%)	1674 (30.6%)	2065 (40.0%)	2103 (42.6%)		1522 (25.3%)	1624 (33.9%)	2696 (56.7%)	
	Missing	110 (0.7%)	53 (1.0%)	36 (0.7%)	21 (0.4%)		22 (0.4%)	40 (0.8%)	48 (1.0%)	
Residency ^#^	Urban	5578 (35.8%)	1610 (29.4%)	2150 (41.6%)	1818 (36.9%)	<0.001	999 (16.6%)	1839 (38.3%)	2740 (57.6%)	<0.001
Education ^#^	Low	6496 (41.7%)	2514 (45.9%)	2042 (39.5%)	1940 (39.3%)	<0.001	3311 (55.0%)	2036 (42.5%)	1149 (24.2%)	<0.001
	Medium	4601 (29.5%)	1630 (29.8%)	1470 (28.5%)	1501 (30.4%)		1751 (29.1%)	1458 (30.4%)	1392 (29.3%)	
	High	3086 (19.8%)	847 (15.5%)	1099 (21.3%)	1140 (23.1%)		603 (10.0%)	846 (17.6%)	1637 (34.4%)	
	Missing	1389 (8.9%)	485 (8.9%)	553 (10.7%)	351 (7.1%)		354 (5.9%)	456 (9.5%)	579 (12.2%)	
Physical activity (MET-hours/week), mean (SD) (*n* = 14,930) ^@^		201.1 (174.1)	212.4 (185.2)	192.3 (169.5)	197.9 (165.4)	<0.001	236.8 (183.0)	202.4 (173.9)	153.7 (149.6)	<0.001
Alcohol consumption ^#^	None	9327 (59.9%)	3247 (59.3%)	3311 (64.1%)	2769 (56.1%)	<0.001	3663 (60.9%)	3002 (62.6%)	2662 (56.0%)	<0.001
	<1/week	1826 (11.7%)	653 (11.9%)	534 (10.3%)	639 (13.0%)		740 (12.3%)	515 (10.7%)	571 (12.0%)	
	1–2/week	1256 (8.1%)	390 (7.1%)	401 (7.8%)	465 (9.4%)		449 (7.5%)	334 (7.0%)	473 (9.9%)	
	3–4/week	705 (4.5%)	220 (4.0%)	216 (4.2%)	269 (5.5%)		264 (4.4%)	178 (3.7%)	263 (5.5%)	
	Daily	1299 (8.3%)	412 (7.5%)	400 (7.7%)	487 (9.9%)		424 (7.0%)	369 (7.7%)	506 (10.6%)	
	Missing	1159 (7.4%)	554 (10.1%)	302 (5.8%)	303 (6.1%)		479 (8.0%)	398 (8.3%)	282 (5.9%)	
Smoking ^#^	Current/ex-smoker	4759 (30.6%)	1606 (29.3%)	1411 (27.3%)	1742 (35.3%)	<0.001	1916 (31.8%)	1379 (28.8%)	1464 (30.8%)	0.002
	Missing	957 (6.1%)	463 (8.5%)	252 (4.9%)	242 (4.9%)		415 (6.9%)	329 (6.9%)	213 (4.5%)	
Body-mass index (kg/m^2^), mean (SD) (*n* = 14,045) ^@^		22.1 (3.1)	22.6 (3.1)	22.0 (3.2)	21.7 (2.9)	<0.001	21.4 (2.7)	22.2 (3.1)	23.0 (3.3)	<0.001
Hypertension ^#^	Yes	1725 (11.1%)	634 (11.6%)	611 (11.8%)	480 (9.7%)	<0.001	466 (7.7%)	529 (11.0%)	730 (15.3%)	<0.001
	Missing	1401 (9.0%)	623 (11.4%)	406 (7.9%)	372 (7.5%)		555 (9.2%)	481 (10.0%)	365 (7.7%)	
Energy (kcal), mean (SD) ^@^		2448.2 (708.4)	2452.8 (750.7)	2212.2 (595.9)	2690.2 (685.5)	<0.001	2597.3 (692.4)	2356.5 (714.5)	2351.9 (689.7)	<0.001
			**Plant-Sourced Nutrient Pattern**				**Animal-Sourced Nutrient Pattern**			
		15,571	5661	4210	5700		6026	4170	5375	
Sex ^#^	Men		2146 (37.9%)	2200 (52.3%)	3280 (57.5%)	<0.001	2435 (40.4%)	1973 (47.3%)	3218 (59.9%)	<0.001
Age in years, median (IQR) ^$^			39.4 (28.2, 55.5)	36.9 (26.8, 49.4)	36.7 (27.1, 47.9)	<0.001	38.6 (27.9, 53.5)	37.3 (27.3, 51.2)	36.7 (26.9, 48.4)	<0.001
Income ^#^	Low		1377 (24.3%)	1147 (27.2%)	2013 (35.3%)	<0.001	2121 (35.2%)	1169 (28.0%)	1247 (23.2%)	<0.001
	Medium		1814 (32.0%)	1414 (33.6%)	1854 (32.5%)		2017 (33.5%)	1381 (33.1%)	1684 (31.3%)	
	High		2415 (42.7%)	1625 (38.6%)	1802 (31.6%)		1852 (30.7%)	1588 (38.1%)	2402 (44.7%)	
	Missing		55 (1.0%)	24 (0.6%)	31 (0.5%)		36 (0.6%)	32 (0.8%)	42 (0.8%)	
Residency ^#^	Urban		2721 (48.1%)	1491 (35.4%)	1365 (23.9%)	<0.001	1708 (28.3%)	1599 (38.3%)	2270 (42.2%)	<0.001
Education ^#^	Low		2014 (35.6%)	1671 (39.7%)	2811 (49.3%)	<0.001	3008 (49.9%)	1684 (40.4%)	1804 (33.6%)	<0.001
	Medium		1592 (28.1%)	1285 (30.5%)	1724 (30.2%)		1673 (27.8%)	1249 (30.0%)	1679 (31.2%)	
	High		1414 (25.0%)	820 (19.5%)	851 (14.9%)		853 (14.2%)	851 (20.4%)	1381 (25.7%)	
	Missing		641 (11.3%)	434 (10.3%)	314 (5.5%)		492 (8.2%)	386 (9.3%)	511 (9.5%)	
Physical activity (MET-hours), mean (SD) (*n* = 14,930) ^@^			165.9 (162.8)	199.1 (167.0)	236.5 (182.5)	<0.001	214.2 (180.6)	197.8 (172.8)	189.2 (166.6)	<0.001
Alcohol consumption ^#^	None		3845 (67.9%)	2422 (57.5%)	3060 (53.7%)	<0.001	3952 (65.6%)	2559 (61.4%)	2816 (52.4%)	<0.001
	<1/week		517 (9.1%)	503 (11.9%)	806 (14.1%)		599 (9.9%)	486 (11.7%)	741 (13.8%)	
	1–2/week		354 (6.3%)	393 (9.3%)	508 (8.9%)		383 (6.4%)	324 (7.8%)	548 (10.2%)	
	3–4/week		206 (3.6%)	197 (4.7%)	302 (5.3%)		222 (3.7%)	188 (4.5%)	295 (5.5%)	
	Daily		419 (7.4%)	376 (8.9%)	504 (8.8%)		398 (6.6%)	312 (7.5%)	589 (11.0%)	
	Missing		320 (5.7%)	319 (7.6%)	520 (9.1%)		472 (7.8%)	301 (7.2%)	386 (7.2%)	
Smoking ^#^	Current/ex-smoke		1344 (23.7%)	1345 (31.9%)	2069 (36.3%)	<0.001	1599 (26.5%)	1199 (28.8%)	1960 (36.5%)	<0.001
	Missing		259 (4.6%)	265 (6.3%)	433 (7.6%)		402 (6.7%)	244 (5.9%)	311 (5.8%)	
Body-mass index (kg/m^2^), mean (SD) (*n* = 14,045) ^@^			22.3 (3.3)	22.3 (3.1)	21.9 (2.9)	<0.001	21.8 (3.0)	22.2 (3.2)	22.4 (3.1)	<0.001
Hypertension ^#^	Yes		781 (13.8%)	449 (10.7%)	495 (8.7%)	<0.001	698 (11.6%)	454 (10.9%)	573 (10.7%)	0.190
	Missing		499 (8.8%)	378 (9.0%)	524 (9.2%)		566 (9.4%)	387 (9.3%)	448 (8.3%)	
Energy (kcal), mean (SD) ^@^			1943.5 (492.3)	2491.4 (491.0)	2917.9 (690.5)	<0.001	2136.7 (624.0)	2385.8 (557.8)	2846.1 (710.9)	<0.001

^#^ Pearson’s chi-squared test; ^$^ Kruskal–Wallis test; ^@^ analysis of variance (ANOVA); T1-tertile 1 (lowest intake); T2-tertile 2; T3-tertile 3 (highest intake); IQR, interquartile range; SD, standard deviation; MET, metabolic equivalent task.

**Table 2:** Selected baseline food and nutrient intake across tertiles of dietary and nutrient patterns among adults 18 years and above, the China Health and Nutrition Survey ^@^.

		T1	T2	T3	*p* Value	T1	T2	T3	*p* Value
		**Traditional Dietary Pattern**				**Modern Dietary Pattern**			
***N***	**15,572**	**5476**	**5164**	**4932**		**6019**	**4796**	**4757**	
**Food Groups, Mean (SD)**									
Rice (g/day)	286.9 (211.4)	100.6 (119.7)	324.1 (137.9)	454.9 (192.5)	<0.001	422.5 (212.2)	212.2 (171.7)	190.7 (146.4)	<0.001
Fish (g/day)	24.1 (47.2)	6.4 (21.0)	20.0 (36.4)	47.9 (65.1)	<0.001	13.8 (33.3)	22.5 (42.4)	38.8 (61.1)	<0.001
Tofu (g/day)	22.7 (42.4)	25.1 (46.0)	24.3 (42.4)	18.4 (37.6)	<0.001	21.4 (45.1)	22.3 (40.3)	24.8 (40.9)	<0.001
Dry tofu (g/day)	10.0 (26.3)	3.1 (15.2)	8.4 (20.9)	19.3 (36.4)	<0.001	10.1 (26.3)	8.7 (24.9)	11.2 (27.5)	<0.001
Fresh vegetable (g/day)	279.1 (179.0)	238.2 (168.0)	256.7 (151.7)	347.8 (196.6)	<0.001	338.4 (204.8)	239.7 (145.7)	243.7 (152.3)	<0.001
Salted vegetable (g/day)	15.7 (46.7)	13.1 (53.7)	13.3 (34.5)	21.1 (48.9)	<0.001	29.6 (68.1)	7.5 (21.6)	6.4 (20.2)	<0.001
Fruit (g/day)	19.4 (72.2)	12.5 (48.0)	20.1 (62.3)	26.3 (98.6)	<0.001	2.2 (15.6)	7.3 (29.5)	53.2 (119.1)	<0.001
Soy milk (mL/day)	5.6 (29.2)	7.5 (36.8)	6.0 (28.0)	2.9 (18.9)	<0.001	0.4 (6.0)	1.3 (9.8)	16.4 (49.8)	<0.001
Milk (mL/day)	5.8 (35.3)	3.5 (28.0)	7.1 (37.2)	6.9 (40.2)	<0.001	0.0 (0.3)	0.1 (2.5)	18.8 (62.0)	<0.001
Milk powder (g/day)	0.4 (5.5)	0.3 (4.2)	0.4 (4.6)	0.4 (7.4)	0.360	0.0 (1.4)	0.1 (2.1)	1.0 (9.6)	<0.001
Whole grain (g/day)	26.7 (82.4)	70.0 (126.2)	5.0 (20.3)	1.5 (11.6)	<0.001	29.8 (101.8)	33.3 (78.4)	16.2 (52.6)	<0.001
**Nutrients**									
Calcium (mg/day)	639.2 (780.3)	608.2 (838.3)	564.1 (641.9)	752.3 (831.3)	<0.001	774.6 (952.5)	543.2 (653.2)	564.7 (614.2)	<0.001
Magnesium (mg/day)	381.8 (239.9)	451.8 (277.9)	318.9 (186.7)	370.1 (222.9)	<0.001	421.4 (283.6)	367.3 (213.3)	346.4 (193.0)	<0.001
Phosphorus (mg/day)	1266.8 (595.0)	1335.5 (669.4)	1083.6 (471.3)	1382.2 (578.1)	<0.001	1378.1 (668.9)	1204.3 (551.0)	1188.8 (511.1)	<0.001
Potassium (mg/day)	2419.5 (2032.4)	2479.1 (2231.7)	2104.9 (1655.9)	2682.8 (2113.4)	<0.001	2858.1 (2464.2)	2127.5 (1693.5)	2159.0 (1597.4)	<0.001
Fiber (g/day)	15.4 (11.4)	18.9 (12.9)	12.6 (9.2)	14.3 (10.7)	<0.001	17.7 (13.2)	14.4 (10.1)	13.4 (9.4)	<0.001
Vitamin A (mg/day)	200.6 (738.4)	106.5 (225.3)	162.1 (353.4)	345.3 (1225.6)	<0.001	106.3 (605.1)	174.5 (504.8)	346.2 (1015.7)	<0.001
Vitamin C (mg/day)	142.1 (178.0)	129.6 (185.8)	129.7 (155.7)	169.1 (187.9)	<0.001	187.5 (214.2)	114.4 (143.0)	112.7 (144.5)	<0.001
Protein (g/day)	73.4 (26.0)	74.9 (28.6)	63.2 (20.9)	82.2 (24.0)	<0.001	71.0 (25.0)	71.3 (25.6)	78.4 (26.8)	<0.001
Fat (g/day)	33.6 (25.0)	26.7 (19.2)	30.2 (22.6)	44.7 (29.1)	<0.001	25.1 (22.2)	30.8 (21.4)	47.1 (26.2)	<0.001
Carbohydrate(g/day)	394.1 (160.9)	437.2 (180.9)	346.7 (127.6)	395.9 (154.8)	<0.001	453.0 (166.6)	383.5 (156.5)	330.2 (127.9)	<0.001
		**Plant-Sourced Nutrient Pattern**				**Animal-Sourced Nutrient Pattern**			
	**15,571**	**5661**	**4210**	**5700**		**6026**	**4170**	**5375**	
**Food Groups**									
Rice (g/day)		242.3 (145.8)	309.8 (202.2)	314.4 (260.1)	<0.001	308.8 (200.3)	278.0 (207.8)	269.4 (223.7)	<0.001
Fish (g/day)		25.3 (43.2)	26.7 (48.6)	20.9 (49.8)	<0.001	16.4 (36.2)	23.4 (44.1)	33.2 (57.8)	<0.001
Tofu (g/day)		18.5 (33.4)	24.1 (42.7)	25.9 (49.3)	<0.001	18.0 (37.8)	23.8 (41.8)	27.1 (47.0)	<0.001
Dry tofu (g/day)		7.0 (18.7)	11.9 (28.7)	11.6 (30.3)	<0.001	6.1 (18.7)	8.7 (22.4)	15.5 (34.2)	<0.001
Fresh vegetable (g/day)		224.0 (130.9)	294.2 (178.9)	322.6 (204.6)	<0.001	278.6 (176.1)	274.4 (181.3)	283.2 (180.4)	0.057
Salted vegetable (g/day)		8.8 (24.7)	12.8 (36.4)	24.8 (65.1)	<0.001	15.7 (42.6)	15.3 (46.7)	16.0 (50.9)	0.740
Fruit (g/day)		21.4 (62.6)	22.5 (74.9)	15.0 (78.5)	<0.001	11.0 (46.8)	17.9 (79.0)	29.9 (87.2)	<0.001
Soy milk (g/day)		7.1 (31.2)	6.1 (29.1)	3.6 (27.0)	<0.001	2.7 (17.9)	4.7 (24.2)	9.4 (40.5)	<0.001
Milk (g/day)		8.0 (40.5)	6.8 (39.4)	2.7 (25.0)	<0.001	1.4 (15.2)	4.4 (28.3)	11.7 (51.8)	<0.001
Milk powder (g/day)		0.4 (4.1)	0.3 (4.5)	0.4 (7.1)	0.930	0.1 (2.2)	0.2 (2.9)	0.7 (8.7)	<0.001
Whole grain (g/day),		8.8 (30.6)	18.9 (54.5)	50.3 (120.5)	<0.001	21.3 (68.3)	32.1 (90.4)	28.7 (89.8)	<0.001
**Nutrients**									
Calcium (mg/day)		280.1 (128.1)	401.9 (182.0)	1171.1 (1081.7)	<0.001	873.1 (1116.6)	454.5 (415.5)	520.2 (360.7)	<0.001
Magnesium (mg/day)		218.9 (64.5)	319.3 (83.4)	589.9 (274.5)	<0.001	394.1 (314.7)	337.5 (165.3)	402.6 (179.9)	<0.001
Phosphorus (mg/day)		816.9 (192.0)	1135.5 (188.3)	1810.5 (628.4)	<0.001	1234.5 (773.8)	1132.4 (385.7)	1407.1 (451.7)	<0.001
Potassium (mg/day)		1271.3 (331.5)	1809.8 (383.1)	4010.3 (2636.8)	<0.001	2900.2 (2912.9)	1920.6 (1116.3)	2267.8 (1006.6)	<0.001
Fiber (g/day)		7.9 (3.0)	12.3 (4.6)	25.1 (13.1)	<0.001	16.2 (13.4)	13.4 (8.4)	16.0 (10.7)	<0.001
Vitamin A (mg/day)		281.7 (1068.4)	186.0 (407.9)	130.8 (470.7)	<0.001	66.8 (131.3)	137.7 (228.3)	399.4 (1207.2)	<0.001
Vitamin C (mg/day)		63.9 (37.6)	94.1 (56.6)	255.3 (249.5)	<0.001	207.8 (249.0)	104.3 (101.5)	97.9 (80.0)	<0.001
Protein (g/day)		55.6 (15.6)	72.9 (17.8)	91.3 (27.0)	<0.001	58.3 (20.6)	69.1 (14.6)	93.5 (25.2)	<0.001
Fat (g/day)		31.6 (21.1)	34.6 (27.1)	34.8 (26.8)	<0.001	19.4 (14.3)	30.4 (15.8)	51.9 (28.7)	<0.001
Carbohydrate (g/day)		271.9 (75.5)	377.8 (85.3)	527.5 (163.9)	<0.001	375.9 (163.0)	378.5 (132.3)	426.6 (173.3)	<0.001

*^@^ p* values were calculated using analysis of variance (ANOVA); T1, tertile 1 (lowest intake); T2, tertile 2; T3, tertile 3 (highest intake).

**Table 3:** Median follow-up time and crude incidence of fractures by tertiles of dietary and nutrient patterns among adults 18 years and above, the China Health and Nutrition Survey (1991–2011).

		T1	T2	T3	Log-Rank Test	T1	T2	T3	Log-Rank Test
	**Total**	**Traditional Dietary Pattern**				**Modern Dietary Pattern**			
***N***	**15,572**	**5476**	**5164**	**4932**		**6019**	**4796**	**4757**	
Median follow-up time (years)	8.9	8.8	8.9	9.0		8.9	9.0	7.0	
Number of fractures	649	220	214	215	0.8441	227	216	206	0.0230
Person-years at risk	162,416.3	54,925.4	52,208.0	55,282.9		63,297.3	54,385.8	44,733.2	
Rate of fracture per 1000 person-years (95% CI)	4.0 (3.7, 4.3)	4.0 (3.5, 4.6)	4.0 (3.6, 4.7)	3.9 (3.4, 4.5)		3.6 (3.2, 4.1)	4.0 (3.5, 4.5)	4.6 (4.0, 5.3)	
		**Plant-Sourced Nutrient Pattern**				**Animal-Sourced Nutrient Patterns**			
***N***	**15,571**	**5661**	**4210**	**5700**		**6026**	**4170**	**5375**	
Median follow-up time (years)	8.9	7.0	9.0	8.9		7.1	9.0	7.1	
Number of fractures	649	198	189	262	0.2531	221	214	214	0.4048
Person-years at risk	162,416.3	46,670.8	51,462.5	64,281.0		59,501.7	51,064.5	51,848.1	
Rate of fracture per 1000 person-years (95% CI)	4.0 (3.7, 4.3)	4.2 (3.7, 4.9)	3.7 (3.2, 4.2)	4.1 (3.6, 4.6)		3.7 (3.3, 4.2)	4.2 (3.7, 4.8)	4.1 (3.6, 4.7)	

CI, confidence interval; T1, tertile 1 (lowest intake); T2, tertile 2; T3, tertile 3 (highest intake).

**Table 4:** Hazard ratios (HRs) and 95% confidence intervals (CI) for tertiles of dietary and nutrient pattern scores and fracture among adults 18 years and above, the China Health and Nutrition Survey (1991–2011).

			HR 95% CI		*p* for Trend	AIC	HR 95% CI		*p* for Trend	AIC
		T1	T2	T3			T2	T3		
Models	Person-Years; Number of Study Participants (Number of Cases)	Cumulative Mean Scores					Overall Mean Scores			
		**Traditional Dietary Pattern**								
Model 1	162,416.3; 15,572 (649)	1.00	1.00 (0.82–1.20)	1.01 (0.84–1.23)	0.887		0.97 (0.80–1.17)	1.10 (0.91–1.33)	0.361	
Model 2	136,542.0; 14,506 (559)	1.00	1.00 (0.82–1.23)	0.99 (0.80–1.22)	0.927		0.98 (0.80–1.21)	1.08 (0.88–1.33)	0.470	
Model 3	130,075.1; 14,193 (540)	1.00	1.05 (0.85–1.30)	1.03 (0.83–1.28)	0.757	9565	1.00 (0.81–1.24)	1.12 (0.90–1.39)	0.313	9564
		**Modern Dietary Pattern**								
Model 1	162,414.3; 15,571 (649)	1.00	1.08 (0.90–1.30)	1.26 (1.04–1.52) *	0.020		1.25 (1.03–1.52) *	1.48 (1.22–1.80) **	<0.0001	
Model 2	136,542.0; 14,506 (559)	1.00	1.05 (0.85–1.29)	1.31 (1.04–1.65) *	0.029		1.25 (1.01–1.55) *	1.59 (1.26–2.01) **	<0.0001	
Model 3	130,075.1; 14,193 (540)	1.00	1.06 (0.85–1.31)	1.34 (1.06–1.71) *	0.019	9559	1.29 (1.04–1.61) *	1.63 (1.28–2.07) **	<0.0001	9550
		**Plant-Sourced Nutrient Pattern**								
Model 1	162,414.3; 15,571 (649)	1.00	0.91 (0.74–1.12)	1.06 (0.86–1.30)	0.487		1.08 (0.89–1.31)	0.95 (0.77–1.17)	0.618	
Model 2	136,540.0; 14,505 (559)	1.00	0.94 (0.75–1.18)	1.08 (0.86–1.36)	0.427		1.11 (0.90–1.37)	0.96 (0.76–1.21)	0.687	
Model 3	130,073.1; 14,192 (540)	1.00	0.94 (0.75–1.19)	1.08 (0.86–1.37)	0.438	9564	1.09 (0.88–1.35)	0.93 (0.74–1.19)	0.551	9563
			**Animal-Sourced Nutrient Pattern**							
Model 1	162,414.3; 15,571 (649)	1.00	1.18 (0.98–1.43)	1.25 (1.02–1.54) *	0.026		1.15 (0.94–1.40)	1.49 (1.22–1.83) **	<0.0001	
Model 2	136,540.0; 14,505 (559)	1.00	1.27 (1.03–1.56) *	1.32 (1.05–1.66) *	0.016		1.18 (0.95–1.47)	1.54 (1.22–1.94) **	<0.0001	
Model 3	130,073.1; 14,192 (540)	1.00	1.29 (1.04–1.60) *	1.37 (1.08–1.72) *	0.008	9557	1.22 (0.98–1.52)	1.61 (1.27–2.04) **	<0.0001	9549
			**Recent Scores**				**Baseline Scores**			
			**Traditional Dietary Pattern**							
Model 1		1.00	0.98 (0.81–1.19)	1.05 (0.87–1.28)	0.600		1.09 (0.90–1.31)	1.06 (0.87–1.28)	0.566	
Model 2		1.00	1.00 (0.81–1.23)	1.04 (0.85–1.29)	0.691		1.07 (0.87–1.31)	1.07 (0.87–1.31)	0.546	
Model 3		1.00	1.02 (0.83–1.27)	1.08 (0.87–1.34)	0.498	9565	1.11 (0.90–1.38)	1.11 (0.90–1.38)	0.337	9564
			**Modern Dietary Pattern**							
Model 1		1.00	1.04 (0.86–1.25)	1.19 (0.98–1.44)	0.083		1.15 (0.95–1.40)	1.20 (0.98–1.45)	0.072	
Model 2		1.00	1.05 (0.85–1.29)	1.18 (0.94–1.48)	0.172		1.19 (0.97–1.46)	1.22 (0.97–1.53)	0.086	
Model 3		1.00	1.02 (0.83–1.26)	1.15 (0.91–1.46)	0.252	9564	1.18 (0.95–1.45)	1.23 (0.97–1.55)	0.084	9562
			**Plant-Sourced Nutrient Pattern**							
Model 1		1.00	1.08 (0.88–1.33)	1.06 (0.83–1.36)	0.664		1.00 (0.82–1.22)	1.22 (1.00–1.49) *	0.037	
Model 2		1.00	1.16 (0.92–1.46)	1.13 (0.86–1.49)	0.411		1.03 (0.82–1.28)	1.27 (1.02–1.58) *	0.027	
Model 3		1.00	1.15 (0.91–1.46)	1.12 (0.85–1.48)	0.455	9564	1.02 (0.81–1.27)	1.24 (0.99–1.54)	0.051	9561
			**Animal-Sourced Nutrient Pattern**							
Model 1		1.00	1.01 (0.83–1.22)	1.04 (0.83–1.29)	0.747		1.04 (0.86–1.26)	1.09 (0.90–1.32)	0.373	
Model 2		1.00	0.97 (0.79–1.19)	0.99 (0.77–1.26)	0.901		1.10 (0.90–1.35)	1.14 (0.93–1.41)	0.209	
Model 3		1.00	0.95 (0.77–1.18)	0.99 (0.77–1.27)	0.909	9565	1.10 (0.89–1.35)	1.18 (0.95–1.47)	0.126	9563

* *p* < 0.05; ** *p* <0.001. AIC, Akaike’s information criterion. T1, tertile 1 (lowest intake); T2, tertile 2; T3, tertile 3 (highest intake). Model 1: Adjusted for sex, age (continuous) and energy intake (continuous). Model 2: Additionally adjusted for educational status (low, medium and high), income (low, medium and high), alcohol consumption (none, <1, 1–2, 3–4 per week and daily), smoking (non-smoker and current/ex-smoker), residency (rural and urban) and physical activity level (metabolic equivalent task-hours/week, continuous). Model 3: Additionally adjusted for body-mass index (continuous) and high blood pressure (yes/no). *p* for trend was obtained by adjusting the tertiles of the pattern scores as a continuous variable.

## Supplementary Materials

The following are available online at www.mdpi.com/2072-6643/9/11/1198/s1, Table S1: Spearman rank correlation coefficients for cumulative factor score means of dietary and nutrient patterns among adults 18 years and above, the China Health and Nutrition Survey by age and sex (1991–2011), Table S2: Median follow-up time and crude incidence of fractures by age and sex categories among adults 18 years and above, the China Health and Nutrition Survey (1991–2011) *, Table S3: Hazard ratios (HRs) and 95% confidence interval (CI) for tertiles of dietary and nutrient patterns and fracture among adults 18 years and above, the China Health and Nutrition Survey by age and sex (1991–2011) ^@^, Figure S1: Nelson–Aalen cumulative hazard estimates by tertiles of A) traditional and B) modern dietary pattern scores (cumulative average) for study participants aged 18 years and over and both sexes (1991–2011), the China Health and Nutrition Study, Figure S2: Nelson–Aalen cumulative hazard estimates by tertiles of: (A) plant-sourced; and (B) animal-sourced nutrient pattern scores (cumulative average) for study participants aged 18 years and over and both sexes (1991–2011), the China Health and Nutrition Survey, Figure S3: Multivariable adjusted hazard ratio (HR) and 95% confidence interval of fractures in joint classified participants across nine strata formed with the tertiles of the modern dietary pattern and animal sourced nutrient pattern, the China Health and Nutrition Survey. Modern I and traditional I was used as the reference. The model was adjusted for sex, age (continuous), energy intake (continuous), educational status (low, medium and high), income (low, medium and high), alcohol consumption (none, <1, 1–2, 3–4 per week and daily), smoking (non-smoker and current/ex-smoker), residency (rural and urban) and physical activity level (metabolic. equivalent task-hours/week, continuous), body-mass index (continuous) and high blood pressure (yes/no). Exposure levels of dietary and nutrient patterns were determined based on cumulative mean, Figure S4: Multivariable adjusted hazard ratio (HR) and 95% confidence interval of fractures in joint classified participants across nine strata formed with the tertiles of the modern dietary pattern and animal sourced nutrient pattern, the China Health and Nutrition Survey. Modern I and animal-sourced I was used as the reference. The model was adjusted for sex, age (continuous), energy intake (continuous), educational status (low, medium and high), income (low, medium and high), alcohol consumption (none, <1, 1–2, 3–4 per week and daily), smoking (non-smoker and current/ex-smoker), residency (rural and urban) and physical activity level (metabolic equivalent task-hours/week, continuous), body-mass index (continuous) and high blood pressure (yes/no). Exposure levels of dietary and nutrient patterns were determined based on cumulative mean, Figure S5: Multivariable adjusted hazard ratio (HR) and 95% confidence interval of fractures in joint classified participants across nine strata formed with the tertiles of the modern dietary pattern and animal sourced nutrient pattern, the China Health and Nutrition Survey. Plant-sourced I and animal-sourced I was used as the reference. The model was adjusted for sex, age (continuous), energy intake (continuous), educational status (low, medium and high), income (low, medium and high), alcohol consumption (none, <1, 1–2, 3–4 per week and daily), smoking (non-smoker and current/ex-smoker), residency (rural and urban) and physical activity level (metabolic equivalent task-hours/week, continuous), body-mass index (continuous) and high blood pressure (yes/no). Exposure levels of dietary and nutrient patterns were determined based on cumulative mean.
