# Supplementary material for: Prospective Associations of Dietary and Nutrient Patterns with Fracture Risk: A 20-Year Follow-Up Study
Source: Nutrients. 2017 Oct 31;9(11):1198. doi: 10.3390/nu9111198 (PMC5707670; doi:10.3390/nu9111198)
Supplement: Supplementary file 1 [file nutrients-09-01198-s001.pdf]

Table S1: Spearman rank correlation coefficients for cumulative factor score means of dietary and nutrient patterns among adults 18 years and above, the China Health and Nutrition Survey by age and sex (1991-2011)

Dietary and nutrient patterns	Traditional dietary pattern Coefficients	P value	Modern dietary pattern Coefficients	P value	Plant-sourced nutrient pattern Coefficients	P value
**Traditional dietary pattern**						
**Modern dietary pattern**	-0.1389	<0.0001				
**Plant-sourced nutrient pattern**	-0.0513	<0.0001	-0.3060	<0.0001		
**Animal-sourced nutrient pattern**	0.1270	<0.0001	0.4617	<0.0001	0.0145	<0.001

Table S2: Median follow-up time and crude incidence of fractures by age and sex categories among adults 18 years and above, the China Health and Nutrition Survey (1991-2011)*

	Males			Females		
	**<50 years**	**>50 years**	**Total**	**<50 years**	**>50 years**	**Total**
**N**	**5,656**	**3,371**	**7627**	**5,789**	**3371**	**7945**
Median follow-up time (years)	9.0	13.7	10.7	7.1	13.9	9.1
Number of fractures	204	107	311	144	194	338
Person-years at risk	53542.7	27534.0	81076.7	51008.7	30330.9	81339.6
Rate of fracture per 1000 person-years (95% CI)	3.8 (3.3, 4.4)	2.9 (3.2, 4.7)	3.8 (3.4, 4.3)	2.8 (2.4, 3.3)	6.4 (5.6, 7.4)	4.2 (3.7, 4.6)

**The analysis did not exclude those cases that had missing values of other covariates (sex, age, energy intake, educational status, income, alcohol consumption, smoking, residency and physical activity level, body-mass index and high blood pressure).*

Table S3: Hazard ratios (HRs) and 95% confidence interval (CI) for tertiles of dietary and nutrient patterns and fracture among adults 18 years and above, the China Health and Nutrition Survey by age and sex (1991-2011)[@]

	Person-years; number of study participants (number of cases)	HR (95% CI)			P for trend
		T1	T2	T3	
**Traditional dietary pattern**					
**Sex**					
Male	62475.2; 6893 (252)	1.00 [reference]	1.29(0.92-1.80)	1.19(0.87-1.63)	0.314
Female	67599.9; 7,300 (288)	1.00 [reference]	0.90(0.68-1.18)	0.91(0.67-1.24)	0.521
**Age**					
<50 years	83634.3; 10342 (293)	1.00 [reference]	1.14(0.85-1.54)	1.10(0.82-1.46)	0.556
>=50 years	46440.8; 6426 (247)	1.00 [reference]	0.96(0.71-1.30)	0.94(0.68-1.31)	0.717
**Modern dietary pattern**					
**Sex**					
Male	62475.2; 6893 (252)	1.00 [reference]	1.18(0.86-1.61)	1.63(1.16-2.30)**	0.006
Female	67599.9; 7,300 (288)	1.00 [reference]	0.96(0.72-1.29)	1.18(0.85-1.65)	0.361
**Age**					
<50 years	83634.3; 10342 (293)	1.00 [reference]	1.10(0.83-1.47)	1.28(0.93-1.77)	0.132
>=50 years	46440.8; 6426 (247)	1.00 [reference]	1.03(0.74-1.42)	1.45(1.01-2.09)*	0.049
**Plant-sourced nutrient pattern**					
**Sex**					
Male	62475.2; 6892 (252)	1.00 [reference]	0.77(0.54-1.10)	0.78(0.55-1.12)	0.269
Female	67599.9; 7300 (288)	1.00 [reference]	1.11(0.82-1.50)	1.41(1.03-1.92)*	0.030
**Age**					
<50 years	83634.3; 10341 (293)	1.00 [reference]	0.87(0.62-1.20)	1.02(0.74-1.42)	0.731
>=50 years	46440.8; 6425 (247)	1.00 [reference]	0.99(0.71-1.37)	1.09(0.78-1.53)	0.610
**Animal-sourced nutrient pattern**					
**Sex**					
Male	62475.2; 6892 (252)	1.00 [reference]	1.63(1.15-2.32)**	1.74(1.22-2.48)**	0.003
Female	67599.9; 7300 (288)	1.00 [reference]	1.13(0.85-1.49)	1.19(0.85-1.65)	0.280
**Age**					
<50 years	83634.3; 10341 (293)	1.00 [reference]	1.48(1.10-1.99)**	1.30(0.94-1.79)	0.113
>=50 years	46440.8; 6425 (247)	1.00 [reference]	1.12(0.81-1.53)	1.47(1.04-2.07)*	0.034

*P<0.05; **p<0.01;

[@] *The model was adjusted for sex, age (continuous), energy intake (continuous), educational status (low, medium and high), income (low, medium and high), alcohol consumption (none, <1, 1-2, 3-4 per week and daily), smoking (non-smoker and current/ex-smoker), residency (rural and urban) and physical activity level (metabolic equivalent task-hours/week, continuous), body-mass index (continuous) and high blood pressure (yes/no). P for trend was obtained by adjusting the tertiles of the pattern scores as a continuous variable. Exposure levels of dietary and nutrient patterns were determined based on cumulative mean.*

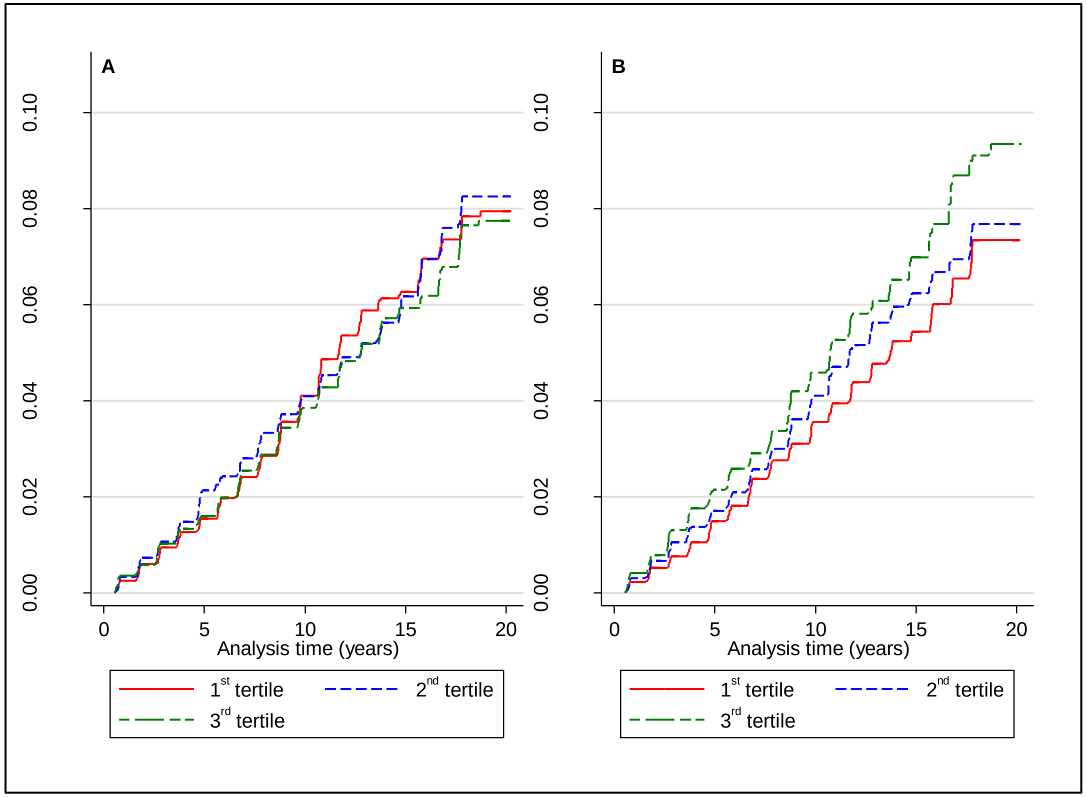

Figure S1: Nelson-Aalen cumulative hazard estimates by tertiles of A) traditional and B) modern dietary pattern scores (cumulative average) for study participants aged 18 years and over and both sexes (1991-2011), the China Health and Nutrition Study

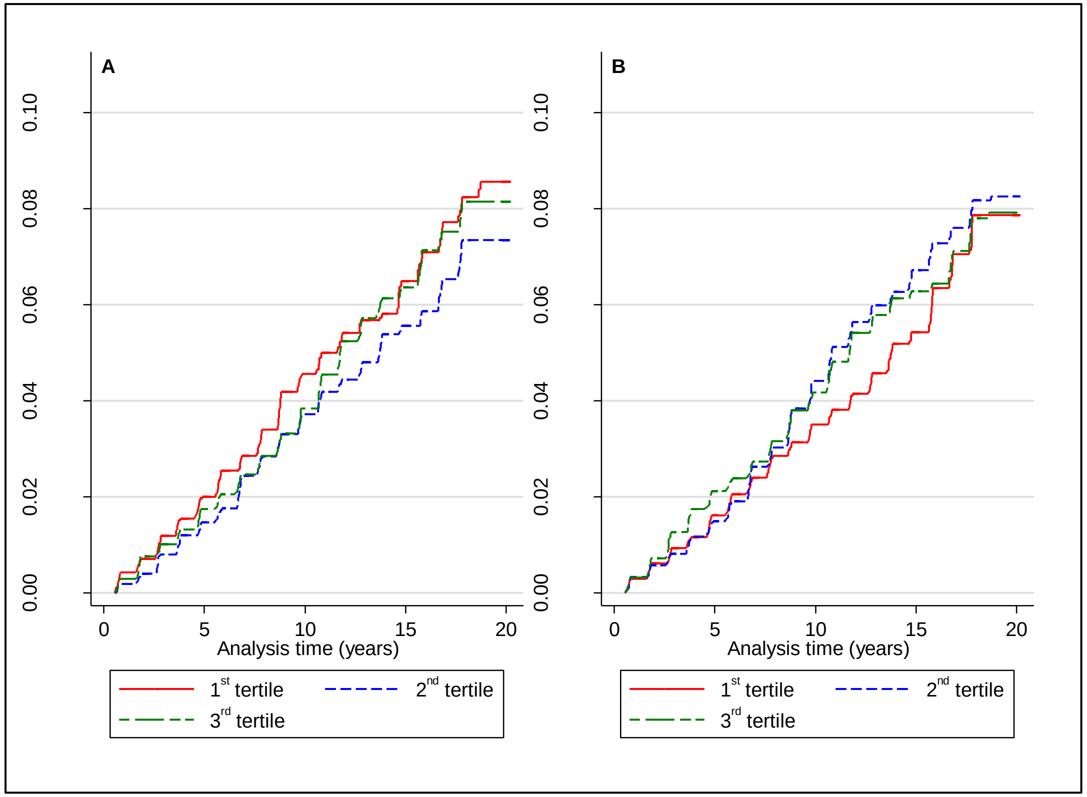

Figure S2: Nelson-Aalen cumulative hazard estimates by tertiles of A) plant-sourced and B) animal-sourced nutrient pattern scores (cumulative average) for study participants aged 18 years and over and both sexes (1991-2011), the China Health and Nutrition Survey

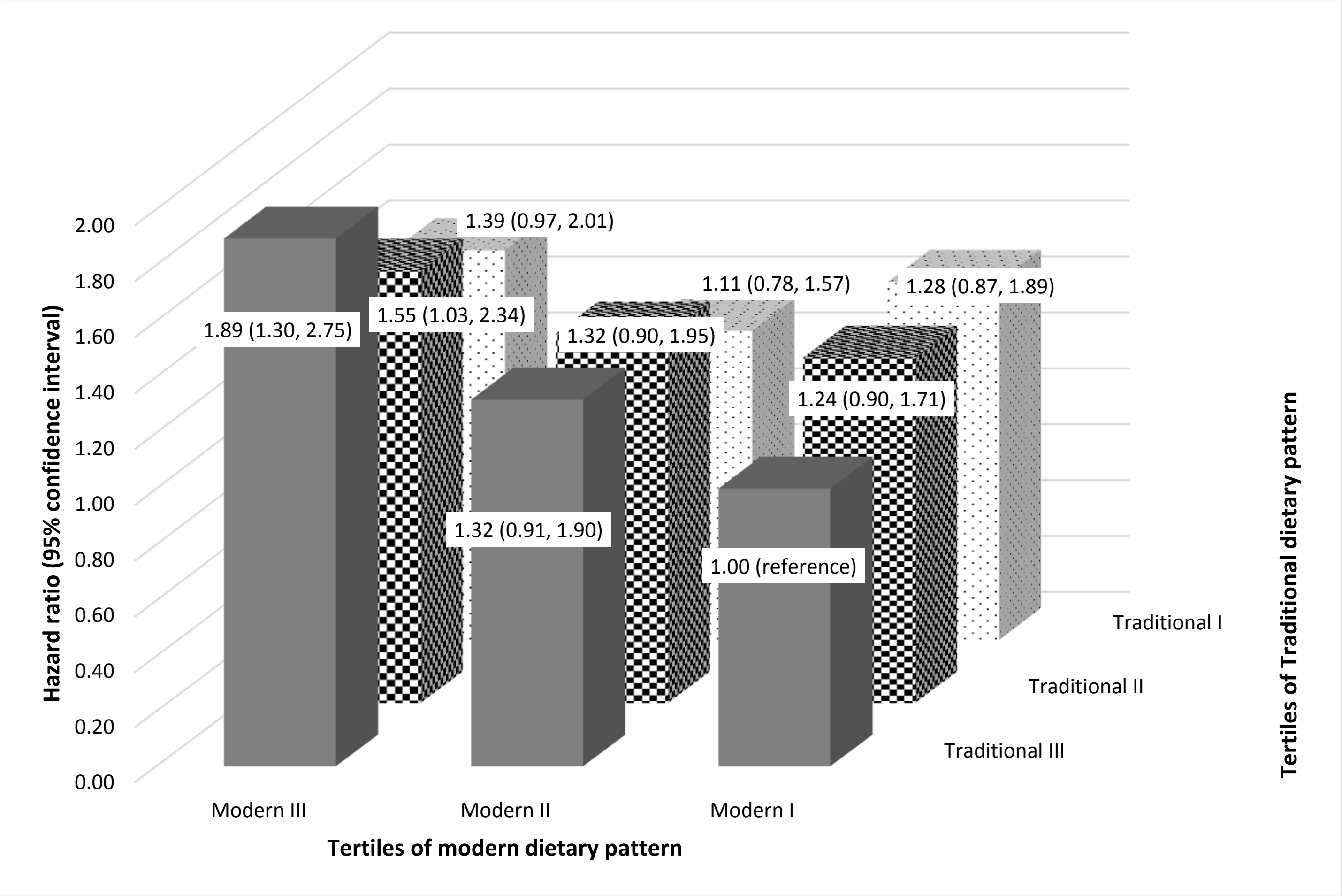

Figure S3: Multivariable adjusted hazard ratio (HR) and 95% confidence interval of fractures in joint classified participants across nine strata formed with the tertiles of the modern dietary pattern and animal sourced nutrient pattern, the China Health and Nutrition Survey. *Modern I and traditional I was used as the reference. The model was adjusted for for sex, age (continuous), energy intake (continuous), educational status (low, medium and high), income (low, medium and high), alcohol consumption (none, <1, 1-2, 3-4 per week and daily), smoking (non-smoker and current/ex-smoker), residency (rural and urban) and physical activity level (metabolic*

equivalent task-hours/week, continuous), body-mass index (continuous) and high blood pressure (yes/no). Exposure levels of dietary and nutrient patterns were determined based on cumulative mean.

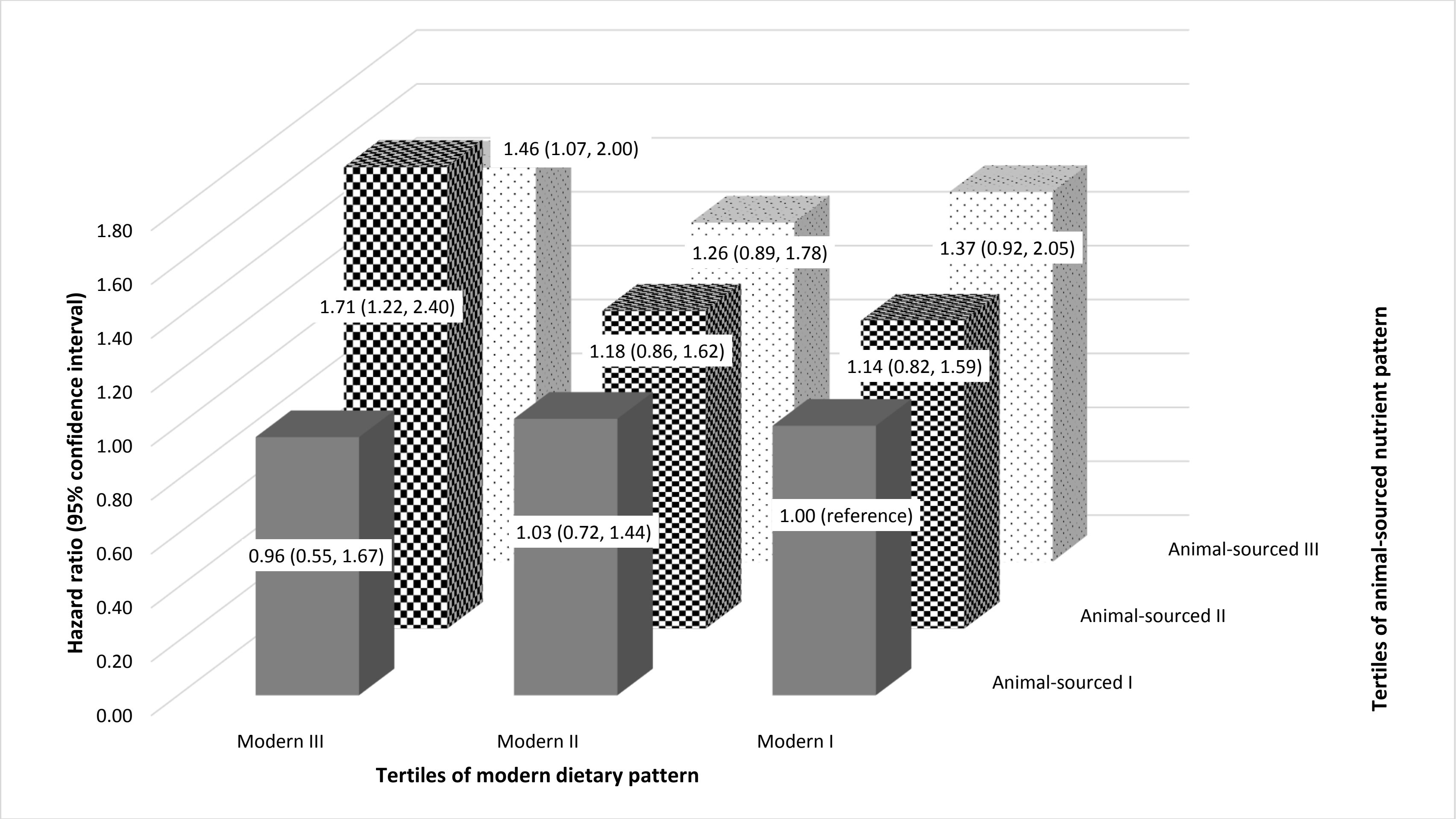

Figure S4: Multivariable adjusted hazard ratio (HR) and 95% confidence interval of fractures in joint classified participants across nine strata formed with the tertiles of the modern dietary pattern and animal sourced nutrient pattern, the China Health and Nutrition Survey. *Modern I and animal-sourced I was used as the reference. The model was adjusted for for sex, age (continuous), energy intake (continuous), educational status (low, medium and high), income (low, medium and high),*

alcohol consumption (none, <1, 1-2, 3-4 per week and daily), smoking (non-smoker and current/ex-smoker), residency (rural and urban) and physical activity level (metabolic equivalent task-hours/week, continuous), body-mass index (continuous) and high blood pressure (yes/no). Exposure levels of dietary and nutrient patterns were determined based on cumulative mean.

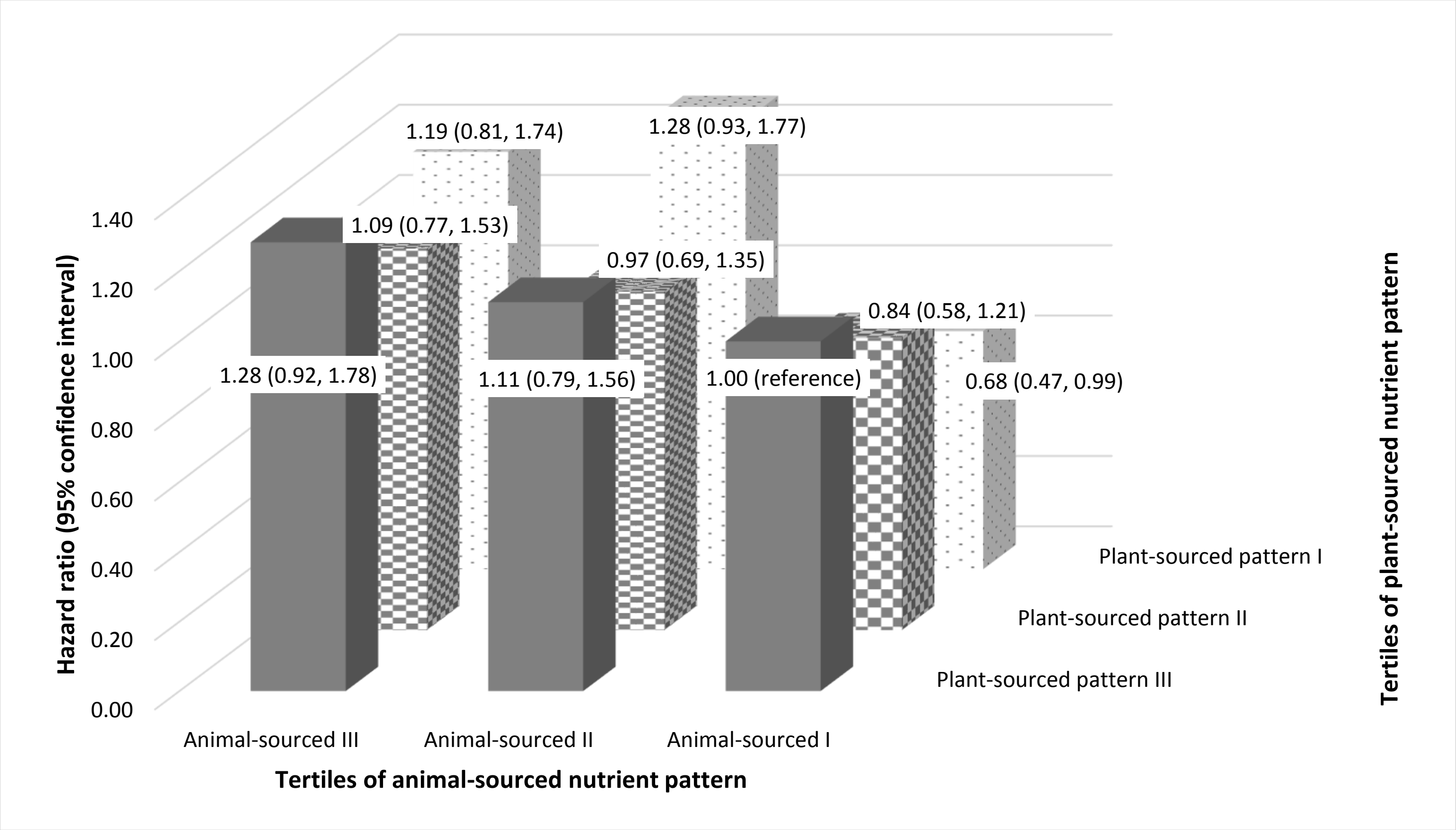

Figure S5: Multivariable adjusted hazard ratio (HR) and 95% confidence interval of fractures in joint classified participants across nine strata formed with the tertiles of the modern dietary pattern and animal sourced nutrient pattern, the China Health and Nutrition Survey. *Plant-sourced I and animal-sourced I was used as the reference. The model was adjusted for for sex, age (continuous), energy intake (continuous), educational status (low, medium and high), income (low, medium and high), alcohol consumption (none, <1, 1-2, 3-4 per week and daily), smoking (non-smoker and current/ex-smoker), residency (rural and urban) and physical activity level*

(metabolic equivalent task-hours/week, continuous), body-mass index (continuous) and high blood pressure (yes/no). Exposure levels of dietary and nutrient patterns were determined based on cumulative mean.
